# Effects of Different Graphic Health Warning Types on the Intention to Quit Smoking

**DOI:** 10.3390/ijerph17093267

**Published:** 2020-05-07

**Authors:** Hyejin Park, Min-Young Hong, In-Seon Lee, Younbyoung Chae

**Affiliations:** Acupuncture and Meridian Science Research Center, College of Korean Medicine, Kyung Hee University, Seoul 02447, Korea; puddle139@naver.com (H.P.); min.hong27@gmail.com (M.-Y.H.)

**Keywords:** smoking, cessation, social threat, advertisements, eye tracking

## Abstract

Anti-smoking advertisements are widely used to demonstrate to smokers the harm of smoking, and graphic health warnings (GHWs) are expected to have a positive effect on the intention to quit smoking. This study investigated which type of GHW (health-related threat (H-GHW) vs. social threat (S-GHW)) is more effective. Two types of GHWs for tobacco were shown to 28 daily smokers and 25 non-smokers while measuring their eye movements using an eye tracker. The time spent fixating on the GHWs was measured as an index of attentional bias. Participants were also asked to evaluate the unpleasantness of the images. They stated their intention to quit smoking in response to each image in a separate session. Multiple regression analysis was used to identify the effects of psychosocial factors on the intention to quit smoking in smokers and the intention to remain as non-smokers in the non-smokers. Both smokers and non-smokers reported greater unpleasantness and cessation intentions in response to H-GHWs than to S-GHWs. Non-smokers found both types of GHWs more unpleasant than smokers did. No differences were found in gaze fixation on GHWs between the two groups. When smokers viewed S-GHWs, the intention to quit smoking was greater as they felt more unpleasant. For non-smokers, the intention to remain non-smokers was greater when they felt more unpleasant and when the attention to H-GHWs was lower. Different psychological factors in anti-smoking advertisements are involved in the intention to quit smoking in smokers and to maintain a non-smoking status in non-smokers. Different approaches should be used according to the types of warning (e.g., warnings emphasizing a negative influence on others or on their own health) in anti-smoking campaigns.

## 1. Introduction

To inform consumers about the risks of smoking, graphic health warnings (GHWs) are required on tobacco product packaging in over 100 countries. Many anti-smoking campaigns aim to elicit quitting behavior by inducing feelings of fear or disgust in smokers [1]. Contemporary campaigns include fear-arousing health-related content, such as images of deformed lungs, oral cancer, and cardiac surgery. These images are likely to evoke negative emotions in both smokers and non-smokers. For non-smokers, carotid surgery or the testimonial of a dying smoker may still cause fear and be undesirable, convincing them to maintain their non-smoking status. Besides fear-arousing visual cues, some anti-smoking campaigns incorporate induced-hypocrisy and empathy-arousing content, which consider consumers’ sociocultural characteristics [2,3]. GHWs include images related to health or social threats. The health threats emphasize the potential physical harm caused if one does not heed the warning, and the social threats highlight the consequent damage to others, thereby arousing one’s ethicality. The effectiveness of GHWs according to the type of image, such as fear-arousing versus empathy-arousing images [2] or fear versus induced hypocrisy [3], has been investigated. Previous studies found that empathy-arousing messages were more effective than fear-arousing messages in anti-smoking campaigns [2], while induced hypocrisy had greater effects than fear appeals did on attitudes toward smoking cessation and intention to quit, at least in Eastern societies [3]. 

The smoking prevalence in South Korea has been estimated via various surveys. The World Health Organization (WHO) reported that about 37.1% of male adults and 5.6% of female adults were smokers in South Korea in 2017, either daily or non-daily. In 2018, the seventh Korea National Health and Nutrition Examination Survey (KNHANES) showed that the cigarette smoking prevalence was 36.7% among male adults and 7.5% among female adults. To reduce the smoking rate, the South Korean government has implemented various anti-smoking regulations such as raising cigarette prices and tax, expansion of smoke-free areas, and banning of tobacco advertisements in stores [4]. Following the recommendation of the WHO’s Framework Convention of Tobacco Control, the Ministry of Health and Welfare of South Korea have modified and complemented GHWs on the cigarette packages over a period of time [5]. Recently, all GHWs were updated to have more impactful images in order to maximize the warning value, considering the familiarity of the GHWs in smokers (2018 December). In addition, the locations of GHWs on cigarette packages have been an important issue. As the duration of eye fixation on GHWs was longer when they were displayed at the top than at the bottom, GHWs would be more effective when located on the top of cigarette packages [6]. In order to estimate the efficacy of renewed GHWs, the current study was conducted with the most recent version of GHWs. 

Since eye tracking captures precise eye movements when an individual is exposed to visual stimuli, it is useful for evaluating the attention paid to GHWs [7]. There is growing evidence of the effects of eye-tracking outcomes with regard to tobacco regulation and communication [8,9,10]. For example, an eye-tracking study explored the visual attention of smokers and found that they preferred the branding and actively avoided the GHWs [11]. The exhibited graphic warnings enhanced visual attention compared to text-only warnings [12,13,14]. Smokers showed more visual attention on warnings with coping text while non-smokers had more visual attention on high-risk information [15]. Furthermore, non-smokers and non-daily smokers showed increased attention to health warnings on plain packs compared to health warnings on branded packs, but daily-smoker’s visual attention to health warning was not affected by how the brand was illustrated on packages [16]. The similar effect was found in adolescent, except that adolescent non-smokers showed biased visual attention to health warnings on both packages plain or branded [11]. These results indicate that warning messages may have different effects on visual attention for smokers compared to non-smokers. As GHWs are supposed to attract attention, it is necessary to measure the attentional behavior of the target consumers (smokers) and non-smokers to better understand their reactions to GHWs and the effectiveness of GHWs.

In this study, we categorized the newly updated GHWs into images of health threats and social threats and examined which type of GHW was more effective in an anti-smoking campaign. We also investigated the selective attention to each type of GHW using eye-tracking equipment and identified the psychosocial factors influencing the intention to quit smoking in smokers and to not start smoking in non-smokers.

## 2. Methods

### 2.1. Participants

The study recruited 28 daily smokers and 25 non-smokers (26 females and 27 males, aged 19–34 years). The smokers had regularly smoked more than five cigarettes daily for over 3 months; the non-smokers had never smoked in their entire lives. The study was conducted at Laboratory of the Cognitive Medical Science, the Department of College of Korean Medicine, Kyung Hee University. Participants were recruited through public advertisements, such as internet communities and public bulletin board with no restriction of age, sex, and occupation. All participants had normal visual acuity, and none had psychiatric or neurological disorders, as confirmed by a Korean medical doctor according to the DSM-IV (SCID I). They received a detailed explanation of the experimental procedure and provided informed consent. This experiment was conducted in accordance with the Declaration of Helsinki and approved by the Institutional Review Board of Kyung Hee University.

### 2.2. Baseline Characteristics

The participants completed questionnaires assessing stress, fear of pain, disgust, empathy, and anxiety. The Stress Response Inventory includes emotional, somatic, cognitive, and behavioral stress responses [17]. The Fear of Pain Questionnaire measures pain-related fear under the assumption that fear is specific to particular stimuli and contexts [18]. The Disgust Scale measures an individual’s responses to various disgust-provoking situations [19]. The Empathy Quotient consists of 60 questions, comprising 40 questions tapping empathy and 20 filler items included to distract the participant from a relentless focus on empathy [20]. The State-Trait Anxiety Inventory assesses how an individual feels “right now”; it may also be used to evaluate feelings at a particular time in the recent past, as well as anticipated feelings either in a specific situation likely to be encountered in the future or in a variety of hypothetical situations [21]. In addition, all of the participants completed the Fagerstrom test nicotine dependence (FTND), and filled out the questionnaire on smoking urges-brief (QSU-Brief) [22].

### 2.3. Two Types of Graphic Health Warnings on Cigarette Packs

**Figure 1:**
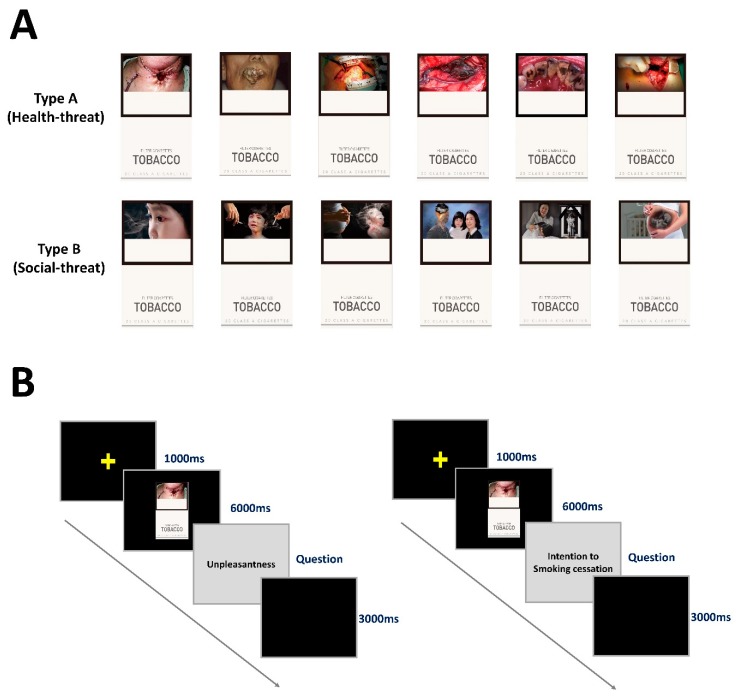
Experimental design and procedures. (**A**): There were 12 visual stimuli, which were cigarette packs with graphic health warnings (GHWs), including health-related threat images (H-GHWs) and social-harm-related images (S-GHWs). (**B**): The unpleasantness of the GHWs images was measured in the first session, and eye movements were recorded simultaneously. The intention to quit smoking in smokers or to remain as a non-smoker in non-smokers was evaluated in the second session.

We used 12 visual stimuli of cigarette packs with GHWs, including images of health risks to self (health-related threat) and others (social-harm-related threat) associated with smoking. The health-related threat images (H-GHWs) were of various diseases caused by smoking, such as cancer, and the social-harm-related images (S-GHWs) emphasized the suffering of others (e.g., family members) due to smoking (Figure 1A). Participants were shown all 12 visual stimuli (6 H-GHWs and 6 S-GHWs), in random order. All of the GHWs were created by the Ministry of Health and Welfare of Korea and are currently on the cigarette packages sold in South Korea. Since 23 December 2016, it became mandatory to include GHWs on the front of cigarette packages, and GHWs have been updated biennially. Ten GHWs have been released by Ministry of Health and Welfare of South Korea every two years, meaning that there are a total of twenty GHWs used in South Korea so far. In this study, we excluded eight images as they were similar to other images, and finally included twelve GHWs. The written warnings and brand names were removed to limit confounding factors.

### 2.4. Psychophysical Assessment

The participants sat in front of a monitor for the experiment, which was divided into two sessions. In the first session, the participants viewed the two types of GHWs in random order and rated the unpleasantness of each image. After 1000 ms for cross fixation, a GHW was presented for 6000 ms at the center of the screen. Then, the participants were asked to rate the unpleasantness of the GHW using a five-point Likert-type rating scale (1 = not unpleasant at all, 5 = very unpleasant). After rating, there was a 3000 ms rest (black screen). In the second session, the participants were shown cigarette packs labeled with the GHWs used in the first session, and the smokers were asked to rate their intention to quit smoking after seeing the picture using a five-point Likert-type rating scale (1 = do not really want to quit, 5 = really want to quit). Non-smokers were asked to rate their intention to remain non-smokers using a five-point Likert-type rating scale (1 = do not really want to remain a non-smoker, 5 = really want to remain a non-smoker). Eye movement was not recorded during the second session, although the cross fixation (1000 ms), experimental stimulus presentation (6000 ms), and rest period (3000 ms) were presented in the same order as in the first session (Figure 1B).

### 2.5. Eye Movement Measurement

During the first session, the subjects’ eye movements were recorded using an eye-tracking system (iView X^™^ RED, SensoMotoric Instruments, Germany). The experimental stimuli were displayed on a 17-in LCD-TFT monitor located approximately 70 cm from the participants’ eyes (visual angles: 16.6° horizontally and 10.8° vertically). For the eye-tracking data, we designated areas of interest (AOIs) within each image to investigate the visual attention according to the type of GHW. We designated the area with the GHW as AOI-A (target AOI) and the rest of the package as AOI-B. The duration of gaze fixation on each AOI was determined, and the percentage of the fixation duration for AOI-A was calculated for each GHW.

### 2.6. Data Analyses

The baseline characteristics of smokers and non-smokers were compared using independent *t*-tests. Behavior and eye tracking data were analyzed using 2 × 2 analysis of variance (ANOVA), and the results are expressed as the mean ± standard error. To test how the intention to quit (not to start) smoking in smokers (non-smokers) might be related to stress, multiple regression models were constructed using the levels of stress, fear of pain, disgust, empathy, anxiety, unpleasant ratings for GHWs (psychophysical rating), and visual attention to GHWs as the independent variables, and the intention to quit smoking as the dependent variable, for each group separately. Statistical analyses were performed using the statistical software package (ver.3.6.0, http://r-project.org) and the Jamovi software (ver. 0.9; http://www.jamovi.org), a graphical user interface to the R. A *p*-value < 0.05 was considered significant. 

## 3. Results

### 3.1. Baseline Characteristics

There were no significant differences in baseline characteristics between the smokers and non-smokers (Table 1).

### 3.2. Psychophysical Rating of Graphic Health Warnings on Cigarette Packs

**Figure 2:**
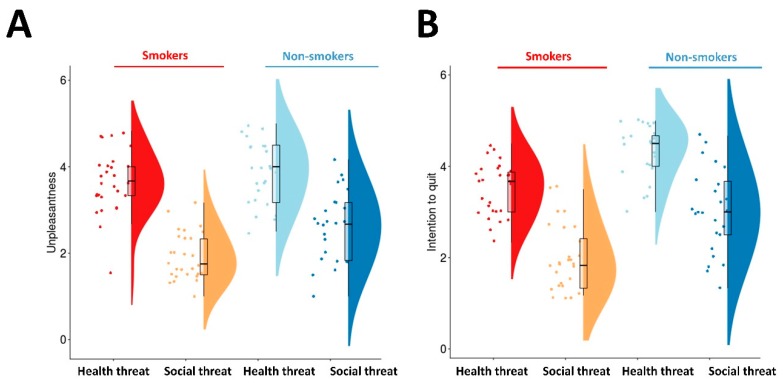
Psychophysical rating of the graphic health warnings on cigarette packs. (**A**): The unpleasantness of the two different types of graphic health warnings (GHWs) was compared between smokers and non-smokers. (**B**): The intention to quit (not start) smoking in response to the two different types of GHWs was compared in smokers (non-smokers). Both groups showed a greater intention to quit smoking or remain a non-smoker in response to H-GHWs than to S-GHWs.

In the first session, the unpleasantness to each GHW image was rated by the participants. We conducted 2 × 2 ANOVA using group (smokers and non-smokers) and type of GHW (H-GHW and S-GHW) as the within-subject factors. This analysis revealed significant main effects of group (F = 11.3, *p* < 0.001) and GHW type (F = 118.36, *p* < 0.001). The interaction (group × type of GWH) was not significant (F = 3.28, *p* = 0.076). Both smokers and non-smokers found H-GHWs more unpleasant than S-GHWs. Non-smokers found both types of GHWs more unpleasant than smokers (smokers: H-GHWs = 3.65 ± 0.14, S-GHWs = 1.92 ± 0.10; non-smokers: H-GHWs = 3.86 ± 0.15, S-GHWs = 2.63 ± 0.16; Figure 2A).

In the second session, the intention to quit smoking evoked by each GHW image was rated. We conducted 2 × 2 ANOVA using the same within-subject factors as those in the above analysis rating unpleasantness. This analysis revealed significant main effect of group (F = 35.0, *p* < 0.001) and GHW type (F = 169.72, *p* < 0.001). The interaction (group × type of GHW) was not significant (F = 0.936, *p* = 0.338). Smokers (non-smokers) showed a greater intention to quit (not start) smoking in response to H-GHWs than to S-GHWs. Non-smokers had a greater intention of maintaining their non-smoking status to any type of GHW compared with smokers (smokers: H-GHWs = 3.51 ± 0.11, S-GHWs = 1.96 ± 0.14; non-smokers: H-GHWs = 4.35 ± 0.12, S-GHWs = 3.01 ± 0.19; Figure 2B).

### 3.3. Visual Attention to Graphic Health Warning Images

**Figure 3:**
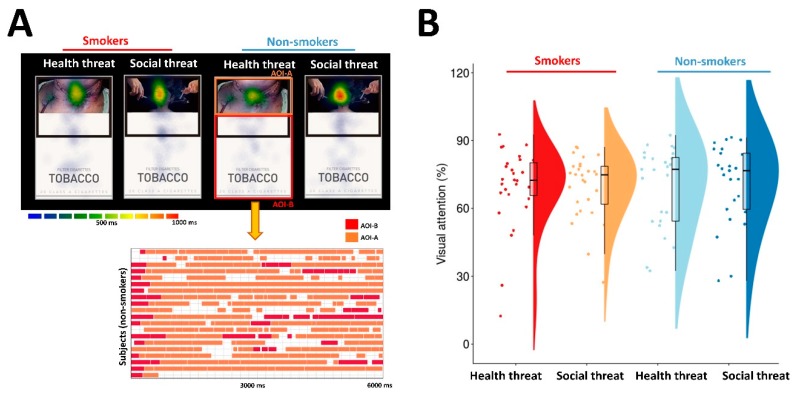
Visual attention to graphic health warnings on cigarette packs. (**A**): Examples of the eye movements in response to graphic health warnings (GHWs) in smokers and non-smokers. (**B**): Visual attention to two different types of GHWs was compared between smokers and non-smokers. There was no significant difference in visual attention between the two different types of GHWs in both smokers and non-smokers.

For the average percentage of eye fixation on AOI-A and AOI-B, we conducted 2 × 2 ANOVA using group (smokers and non-smokers) and GHW type as the within-subject factors. The results revealed no significant main effect of group (F = 0.241, *p* = 0.625) or GHW type (F = 0.074, *p* = 0.787) or group × GHW type interaction (F = 0.106, *p* = 0.746). There was no significant difference in visual attention between the two different types of GHWs in both smokers and non-smokers (smokers: H-GHWs = 69.2 ± 3.4, S-GHWs = 69.6 ± 2.7; non-smokers: H-GHWs = 69.8 ± 3.7, S-GHWs = 70.9 ± 3.6; Figure 3). 

### 3.4. Multiple Regression Analysis

Many psychosocial factors and visual attention can be important factor of smokers’ intentions to quit smoking and non-smokers’ intentions to maintain their non-smoking status. We conducted two multiple regression analyses to reveal psychosocial factors involved in intentions to quit smoking in smokers and factors involved in intentions to maintain a non-smoking status in non-smokers, separately. 

Multiple regression analysis of smokers’ intentions to quit smoking after viewing the S-GHWs revealed that the unpleasantness of S-GHWs (β = 0.560) significantly contributed to their intention to quit smoking (*R*^2^ = 0.557). By contrast, no factors contributed to smokers’ intentions to quit smoking in the case of H-GHWs. 

Multiple regression analysis of non-smokers’ intentions to maintain their non-smoking status after viewing the H-GHWs revealed that the unpleasantness of the H-GHWs (β = 0.622) and attention to H-GHWs (β = −0.442) significantly contributed to their intention to remain non-smokers (*R*^2^ = 0.487). By contrast, no factors contributed to their intention to remain non-smokers in the case of S-GHWs (Table 2).

## 4. Discussion

This study investigated the effectiveness of GHW labels and explored selective attention according to the contents. The psychophysical rating indicated that the H-GHWs were more unpleasant and increased smokers’ intentions to quit smoking compared with the S-GHWs. However, no differences were found in the visual attention to GHWs between the smokers and non-smokers. To explore the psychosocial factors involved in the intention to quit smoking, we performed multiple regression analysis. The results demonstrated that (1) when smokers viewed S-GHWs, the intention to quit smoking was greater as they found them more unpleasant, and (2) when non-smokers viewed H-GHWs, the intention to remain as non-smokers was greater, as they found them more unpleasant, and as they showed lower visual attention. 

In this study, unpleasant GHWs predicted the intention to quit smoking in smokers when they viewed S-GHWs, but not H-GHWs. Comparing the effectiveness of anti-smoking public service announcements that arouse fear versus empathy, empathy-arousing messages were more persuasive, perhaps because fearful messages tend to activate, whereas empathy appeals tend to mitigate, psychological reactance [2]. Another study also suggested to use content inducing a moderate level of fear in anti-smoking advertisements, since excessive fear might lead to avoidance of the message, whereas a low level of fear might fail to catch the attention of the viewers [23]. Moreover, as the contents of the S-GHWs are related to induced-hypocrisy and empathy, these images might force viewers to reconsider their sociocultural characteristics. According to the previous study, smokers could be categorized into “independent self” and “interdependent self” according to the self-construal tendency. Their intentions to quit, in response to anti-smoking advertisements, were stronger in interdependent group than in independent group [3]. Therefore, it can be assumed that interdependent smokers are more easily persuaded by empathy-arousing content, and unpleasant S-GHWs may increase the intention to quit smoking more strongly compared with any other stimulus.

In this study, non-smokers had a greater intention to maintain their non-smoking status when they felt more unpleasant after they viewed H-GHWs, however, this relationship was not found in the S-GHWs trials. Smokers had a greater intention to quit smoking when they felt more unpleasant and had lower visual attention to H-GHWs. For non-smokers, the tendency to avoid material arousing disgust and fear could have led to the lower visual attention, which is related to their intent to maintain their non-smoking status. In previous studies, smokers exhibited active avoidance of GHWs via top-down voluntary control of attention [11]. Moreover, smokers showed delayed attentional orientation responses and reduced and delayed emotional responses [24]. The present study found that non-smokers also displayed avoidance behavior related to the intention to quit smoking.

Smokers had a greater intention to quit smoking when the S-GHWs were more unpleasant. By contrast, non-smokers had a greater intention to maintain their non-smoking status when the H-GHWs were more unpleasant and their visual attention to H-GHWs was lower. Our results suggest that it might be helpful to consider psychological factors which are related to smokers, the main target of health warnings (e.g., unpleasant to social threat warnings) in future anti-smoking campaigns. For smokers, GHW labels involving content that forces viewers to consider their sociocultural characteristics should be designed and updated regularly. On the other hand, for non-smokers, H-GHWs seem to be effective at maintaining their non-smoking status. Further study is needed to prove the effectiveness of the different approaches mentioned above. Notably, bias towards branding rather than health warning in smokers were not influenced by the familiarity with health warnings [11]. However, the familiarity with the GHWs might be different between smokers and non-smokers in the present study, and it may affect their responses. The factor of familiarity with health warnings should be considered to understand the different behaviors between smokers and non-smokers in future studies.

Pharmacological aids, including nicotine replacement therapy or varenicline, have been used for smoking reduction and cessation [25]. The electronic cigarette is a relatively new smoking cessation aid, and recent studies have shown that it is more effective in smoking cessation than nicotine replacement therapy [26,27]. Furthermore, e-cigarettes are more cost-effective than nicotine replacement therapy [28]. However, the issue of electronic cigarettes has been one of the most controversial topics in public health, and the main issues are their safety and the significance of their effectiveness as smoking reduction intervention [29]. In addition to these smoking cessation aids, health warnings on tobacco packages, which is one of the most direct methods of communicating with smokers, might be effective to contribute to smoking reduction and cessation. 

There are some limitations to this study. First, our findings are mainly from relatively young participants in their twenties or thirties. Since there are many older smokers, it is necessary to evaluate older subjects to generalize our findings. Second, since we compared the effects of two types of GHWs on the intention to quit smoking, the text of the warning was not included in the visual stimuli. Real anti-smoking campaigns use text warnings together with graphics to deliver stronger messages. GHWs attract more attention and result in greater recall of the health messages than text-only warnings [7]. It would be interesting to compare visual attention and the intention to quit smoking after viewing text-only, graphics-only, and combined graphics with text health warnings in further studies. The text warnings and brand names were removed to limit confounding factors in this study. However, both graphic and text warnings on cigarette packs might contribute to smokers’ intentions to quit and non-smokers’ intentions to remain as non-smoker. The effects of graphic and text warnings on smoking related behaviors, as well as their interaction, should be investigated in the future. The sample size in each group was very limited to conduct the multiple regression model in the present study. In order to draw more concrete conclusion on different effect of GHW types, it will be necessary to include larger sample size in the future study. Lastly, we did not divide smokers into two groups (independent-self or interdependent-self group) based on the self-construal theory. In order to investigate the influence of sociocultural characteristics of smokers on their responses to health warnings, it will be necessary to include more smokers to categorize the smokers’ self-construal tendency. 

## 5. Conclusions

In conclusion, we found that psychological factors, which are related to different graphic health warnings, are involved differently in the intentions to quit smoking by smokers and in the intentions to maintain a non-smoking status in non-smokers. Unpleasantness of S-GHWs may be a crucial factor for smokers’ intentions to quit smoking while unpleasantness of H-GHWs may be an important factor for non-smokers to remain as non-smokers. Since smokers are the key target of GHWs, the psychosocial factors which are selectively related to smokers, such as the relationship between social threat and unpleasantness, should be extensively studied.

## Figures and Tables

**Table 1:** Baseline characteristics of the smokers and non-smokers.

	Smokers (*n* = 28)	Non-Smokers (*n* = 25)	Significance
Females, *n* (%)	13 (46.4%)	13 (52.0%)	
Age	22.7 ± 0.5	24.0 ± 0.7	*p* = 0.141
FTND	2.9 ± 0.4	*n*/a	
QSU-Brief	33.0 ± 1.9	*n*/a	
SRI	31.0 ± 4.0	26.4 ± 4.4	*p* = 0.429
FPQ	82.3 ± 2.6	87.4 ± 3.3	*p* = 0.216
DS	48.5 ± 2.0	53.6 ± 2.3	*p* = 0.092
EQ	43.7 ± 2.0	41.1 ± 2.2	*p* = 0.372
STAI	41.5 ± 0.9	39.0 ± 1.5	*p* = 0.309

FTND, Fagerstrom test nicotine dependence; QSU-Brief, questionnaire on smoking urges-brief; SRI, Stress Response Inventory; FPQ, Fear of Pain Questionnaire; DS, Disgust Scale; EQ, Empathy Quotient; STAI, State-Trait Anxiety Inventory; *n*/a, not applicable.

**Table 2:** Multiple regression analysis of the intentions to quit smoking by smokers and to maintain a non-smoking status in non-smokers.

**Smokers**	**Health Threat**			**Social Threat**		
	**Standardized Coefficient (β)**	***t***	**Significance**	**Standardized Coefficient (** **β)**	***t***	**Significance**
Constant		3.028	0.007		−0.532	0.601
SRI	−0.068	−0.289	0.776	−0.145	−0.728	0.475
FPQ	0.086	0.364	0.720	0.232	1.165	0.258
DS	0.156	0.592	0.560	0.072	0.262	0.795
EQ	–0.466	−1.964	0.064	−0.021	−0.097	0.923
STAI	−0.164	−0.641	0.529	0.072	0.338	0.739
Unpleasantness of GHWs	0.227	1.055	0.304	0.560	2.127	0.046 *
Attention to GHWs	−0.107	−0.552	0.587	0.021	0.113	0.911
Model fit	R^2^ = 0.301			R^2^ = 0.557		
**Non-smokers**	**Health Threat**			**Social Threat**		
	**Standardized Coefficient (β)**	***t***	**Significance**	**Standardized Coefficient (** **β)**	***t***	**Significance**
Constant		3.584	0.002		−0.062	0.951
SRI	−0.087	–0.351	0.730	0.070	0.214	0.833
FPQ	−0.412	−1.307	0.209	0.358	0.957	0.352
DS	−0.189	−1.021	0.322	−0.154	−0.444	0.663
EQ	−0.068	−0.247	0.808	−0.008	−0.032	0.975
STAI	0.263	1.118	0.279	0.085	0.270	0.790
Unpleasantness to GHWs	0.622	3.074	0.007 *	0.093	0.342	0.736
Attention to GHWs	−0.442	−2.107	0.050 *	0.363	1.449	0.165
Model fit	R^2^ = 0.487					

* Significant factors related to the intention to quit smoking among smokers. SRI, Stress Response Inventory; FPQ, Fear of Pain Questionnaire; DS, Disgust Scale; EQ, Empathy Quotient; STAI, State-Trait Anxiety Inventory; GHW, graphic heath warning.

## Data Availability

Not applicable.
